# Metropolitan Hospital Practice

**Published:** 1828-04-01

**Authors:** 


### METROPOLITAN HOSPITAL PRACTICE.

### 1. COLDSTREAM GUARDS HOSPITAL.

#### DISEASE OF THE HEART.

We shall present to our readers a short statement of this case (from the Medical Gazette) before we make any comment.

A young soldier (aged 23) was received into hospital on the 17th of November, for feverishness and constipation, which seemed to give way in three days. On the 26th he had a fresh attack. On the 1st of December Mr. Maynard saw him. "He appeared very feverish, anxious, and irritable; *had a pulse of very unusual and remarkable strength; it was not only visible at the wrist, but in the fleshy parts of the thighs and belly;* his skin hot, with great thirst." He was bled to *ten ounces* from the arm, without any relief, or any sensible effect on the pulse. From this time till the 10th December he varied much; but, at the latter period, complained of pains in his knees, breast, shoulders, and bowels. On the 23d the fever was renewed, and calomel and opium were given at night, without benefit. A troublesome cough was now added to the other symptoms. There was still no suspicion of disease of the heart.

"On the 25th, however, there was something in the stroke of the pulse which it is impossible to describe, what I never myself felt before, combined with a thrill, almost a hiss, under the finger, more like the vibration of a harp-string; it was very remarkable in the right wrist, but still more so in the left. *The heart was felt to be beating short, quick, and violently,* 130 *in a minute.* On the application of the finger to the space between the second and third rib, a something between a crepitus of air in cellular membrane, and the thrill at the wrist could be discerned at every pulsation, *that showed us pretty clearly the real complaint.* The stethoscope was applied, and the preternatural rushing sounds very clearly heard. About 16 ounces of blood were taken from the arm; it was cupped, but not much, for the coagula were not firm, nor was the buff very marked, being grey rather than the usual yellow. I felt his pulse that night while he slept, and it was comparatively quiet and soft, having lost all that hard jerking vibration it had in the morning. 26th and 27th—Much the same, pulse less violent, and No. 122. On the 28th all the bad symptoms increased; he was restless, anxious, and evidently weaker; had no sleep, felt pain, expressed a wish for nourishment, but complained he could not eat; his pulse was regular and less strong, but had the same remarkable jerk and thrill. Towards the morning of the next day he asked for the close-stool; he refused the bed-pan, and got out of his bed and used it. In half an hour after he had laid himself down again, he died without any struggle.

"On the 31st we examined the body. On raising the sternum, the heart lay more exposed to view than usual, less enveloped in the lungs, of enormous size, and occupying a more horizontal position in the chest. There was no blood whatever in the cavities of the chest or pericardium, but the serous fluid in the pericardium was in double quantity. On looking attentively at the heart as it lay, there appeared, between the origin of the pulmonary artery and the arch of the aorta, a small tumour, of livid colour, having a surface of pericardium, but evidently going into ulceration. It felt solid, but not hard. The right ventricle was very thin and flabby, the left as remarkably thick and firm and muscular. The vessels were all in their natural state, neither larger nor smaller nor discoloured. The interior of the right ventricle was natural. At the upper part of the septum ventriculorum, just where the edges of the semilunar valves mark the origin of the aorta, an orifice appeared, through which was escaping grey flakes of coagulated lymph, which were followed by clots of black blood, and some in a fluid state. This tumour, when emptied, would contain a pigeon's egg; the orifice was ragged, and the interior surface rough and fibrous, like the cut surface of the heart; it was not thin, nor pervious to blood in any part. Two of the semilunar valves were destroyed, one was entire. Quære. What was the cause of death in this case?"*

The foregoing case offers food for much reflexion. We would ask Mr. Maynard, in the first place, how it was that the state of the vascular system, on the 29th November, did not excite his attention to the state of the central organ of the circulation? He examined the patient's abdomen, and found the pulse *visible* there —yet he did not examine the chest. Secondly, was the detraction of ten ounces of blood from a young grenadier, with the above-mentioned phenomena, a sufficient depletion? Thirdly, when he says, on the 25th, that a something between a crepitus and thrill felt in one of the intercostal spaces, "*shewed pretty clearly the real complaint*," we would ask him what was the real complaint then, and how it was known by the foregoing phenomena, as we are not aware that the said phenomena afford any data for a fixed diagnosis? We do not ask these questions from any captious or hypercritical motives; but to shew Mr. Maynard and the public, that auscultation is not sufficiently attended to. The complaint, in this case, was not suspected till within three days of the patient's death, and even then, we question whether a proper diagnosis was formed. The extent over which the heart beat—the impulsion—the difference of impulsion in the two sides of the heart, were not investigated, (at least there is no mention of them,) yet on them would chiefly have rested the diagnosis, even on the first day the patient entered the hospital. In respect to the query at the end of the case—we conceive that there can be very little difficulty in concluding that immense hypertrophy of the left side of the heart—dilatation of the right chambers—valvular disease—and a tumour of the heart going into "ulceration," were quite enough to destroy the life of even a grenadier of the guards. In respect to the nature of this tumour, we have no doubt that it was a false aneurism, or aneurismal pouch going off from between the heart and aorta, such as we have described, in several instances, in the 15th number of this Journal. Considering the obloquy which has been attempted to be thrown on auscultation, of late, we have deemed it necessary to comment on the foregoing case, in order to shew the value of the auscultic diagnosis, and the danger of neglecting it.

* Med. Gazette, No. 6. p. 163.

---

### 2. ST. GEORGE'S HOSPITAL.

### Bleeding in Erysipelas.*

The patient, a soldier, æt, 38, was admitted Nov. 24th, 1827, in consequence of a lacerated wound on the left side of the forehead, and contusion behind the ear, which he had met with four days previously. There was a good deal of action in the system, with head-ache and costive bowels, for which he was bled to ℥ xxx. and took cal. grs. ij. p. jal. grs. vj. 4tis horis. On the next day, he was bled again to ten ounces, and ordered salines with sulphate of magnesia. On the 26th, he had much pain in the head—general excitement—pupils sluggish, and an erysipelatous blush appeared upon the forehead. *Lot. spt. fronti. V.S. ad* ℥xv. The erysipelas extended with thirst, head-ache, hard pulse, and he was again bled to twenty ounces on the 27th. Next day the erysipelas had extended over the right

* Med. Gazette. No. 6. Jan. 12

cheek, with semi-delirium—quick and full pulse—constant nausea. The blood drawn on the preceding day was covered with a thick layer of transparent fibrine. *Rep. V.S. ad* ℥xx. *Cont. med.* Instead of twenty, upwards of thirty ounces of blood were taken by mistake, but during the bleeding the erysipelatous blush faded, and never afterwards returned, at least so distinctly as before. The wounds on the head healed, and, in the beginning of Jan. the patient left the hospital cured.

Not very long ago, the surgeons of St. George's Hospital were accused in the Lancet, and that in pretty broad terms, of killing their patients, by *invariably* treating them, when labouring under erysipelas, with bark! The case above narrated forms, as our cotemporary justly observes, an apt illustration of the *veracity* of that statement.

### POPLITEAL ANEURISM IN BOTH LEGS—SECONDARY HÆMORRHAGE.*

This case, we understand, excited much interest at the time, and furnished food for no little misrepresentation and vituperation in a certain quarter.

The patient was a carrier, æt. 53, admitted August 1st, with a large aneurism in the left ham, which had commenced twelve months previously. The tumour had made considerable progress, and was in one part exceedingly near the surface, but not the least discolouration of the integument was present. His health had been always good, until the commencement of the disease, at which time he was much harrassed in body and mind by a trial at law, since which he has been very nervous and irritable. On his admission, he was labouring under a good deal of fever, with great action in the pulse and vascular system generally. On inspecting the other ham, a distinct aneurismal tumour was discovered pulsating with extreme violence, and on the right side of the neck the carotid was seen beating very forcibly. Of the existence of the second aneurism the patient was not aware, and he was accordingly left in ignorance of it. He was kept quiet and bled until the 9th, when the operation was performed in the usual manner. The ligature separated on the 23d, (fourteen days after its application,) and at this time the wound was little disposed to heal, and the thigh was much swollen. On the 6th September, (thirteen days after the separation of the ligature,) secondary hæmorrhage took place from the wound—the bleeding could not be entirely checked by the tourniquet, &c. and on the morning of the 8th, Mr. Brodie tied the artery at the groin. This ligature remained until the 29th, (twenty-one days,) and on the 11th October, (twelve days subsequently,) secondary hæmorrhage, to the extent of a pint and a half, took place from the upper wound. The patient was now in that state of irritability and excitement, that any further operation was almost out of the question, and, indeed, he positively declared that he would undergo no more. Accordingly, pressure, by means of properly adapted trusses, pads, &c. was carefully employed, but only with temporary effect, for on the 30th bleeding took place, and, from that time until the 2d November, when he died, blood was continually oozing from beneath the compresses, although gentlemen were constantly sitting by the poor fellow's side, day and night. The immediate cause of death was mortification, or rather a condition more nearly approaching to "dry gangrene" of the limb.

On dissection, it was found that the ligature in the groin had been applied on the superficial femoral, immediately below the giving off of the profunda. The artery at this point was entirely destroyed by ulceration, so that no clot or adhesions were discovered, and from this spot to the site of the original operation both artery and vein were converted into a ligamentous undistinguishable mass. The aneurismal tumour in the ham was solid, no larger than an orange, and the vessels passing to and from it obliterated. In the other ham was a good sample of incipient aneurism.

We could make some remarks upon this case, did our limits permit. As it is, we shall content ourselves with copying from the Gazette, with a slight alteration or two, the contradictions of the erroneous statements which have gone forth.

*Lancet.*—"Tumour remarkably tense and solid."

*The fact.*—It was soft, and could be almost entirely emptied by pressure.

* Medical Gazette, No. 7.

*Lancet.*—" Afforded rather an indistinct pulsation."

*The fact.*—The pulsation was visible two or three beds off.

*Lancet.*—" Integuments about to ulcerate."

*The fact.*—Not even a speck of discolouration had appeared.

*Lancet.*—" The man was kept *at least* a fortnight in the hospital before the artery was tied."

*The fact.*—He was admitted on the 1st, and the operation was performed on the 9th. It was not done sooner, because the patient had much fever, and required repeated blood-letting and purging.

*Lancet.*—" The operation should have been performed immediately on the patient's admission."

*The fact.*—It should not.

*Lancet.*—" Mr. Brodie attempts to pass a *straight* probe by main force *under*, for we cannot call it *round* the artery."

*The fact.*—Mr. Brodie attempts to do no such thing. He uses the eye-probe, simply because it is more flexible than the common aneurismal needle, and admits of any curve required. The operation was performed with great facility.

Now if this hospital report of the Lancet's be not a tissue of the most palpable blunders, we know not what is. We have one or two cases by us at the present moment, which will teach the Lancet what sort of stuff the " *surgical capability*" of its reporters is made up of.

---

### 3. MIDDLESEX HOSPITAL.

SECONDARY HÆMORRHAGE.*

J. Willmott, æt. 37, prompter of Drury-lane Theatre, had his left leg removed, by amputation above the knee, for caries of the tarsus, on the 10th October. The case apparently did well, the ligatures came away on the 20th, and the stump had healed, save that two small sinuses remained leading towards the bone. He left the hospital on the 18th November, and resumed his employment at the theatre, one sinus still remaining open, and the bone appearing likely to exfoliate. On the 16th December, the stump swelled and grew more painful, and on the 19th, a violent hæmorrhage took place, which was arrested by a ligature on the limb. He was again brought to the hospital, when Mr. Mayo tied the common femoral artery, distinctly *above* the origin of the profunda. On tying the ligature, the lower part of the artery continued to pulsate, though not so strongly as before. Mr. Mayo thinking that the knot might have slipped, applied a second ligature, but still the pulsation remained. On removing the bandages from the stump, there existed a sinus in the direction of the femoral artery, and a second opposite the bone, and through them the blood must have issued. No more hæmorrhage ensued, but it was necessary to remove some dead bone, and on the 1st January, the ligature had not separated from the vessel.

T. Bailly, æt. 58, had his right leg amputated above the knee, for ulcer and caries of the bone, on the 13th November, by Mr. Mayo. The vessels bled furiously, and sixteen were tied, but in the evening secondary hæmorrhage came on, and it was necessary to secure three more. Next day there was great nervous irritation, twitchings of the stump, &c. from which he rallied for a time, but sank on the 1st January. One ligature, in the direction of the main artery, had not separated, and from this spot hæmorrhage had occurred which was restrained once by pressure, and on its recurrence by the actual cautery. Upon examination, the bleeding was found to have proceeded from a small artery upon the vastus internus, the femoral being firmly closed, and containing a clot, an inch in length. The plugging up of the vessel was found to have depended on the adhesion of the outer coat only, the inner coat not having entered into the cicatrix in the manner described by Dr. Jones. This appearance is very well shewn in a wood-cut.

Between these two cases, though similar in some respects, there is yet an obvious difference. In the first, there was no particular hæmorrhagic tendency at the time, and all went on favourably enough, save that from weakness of constitutional power, or other cause, a sinus remained, and the ulceration sub-

---

* London Med. Gazette, No. 6, Jan. 12.

sequently extended to the artery. In the second case, the hæmorrhagic disposition was marked, and the patient seems to have sunk more from the effects of what took place on the day of operation, than from the trifling bleeding which occurred subsequently, though this, no doubt, was the cause of great irritation to the system. We have heard a comparison drawn between Mr. Mayo's first case, and that related above from St. George's Hospital, but, to our minds, a most inappropriate and unfair one. In the one case, there was disease of the artery in a decidedly aneurismal habit—in the other, there was nothing of the kind.

---

### 4. ST. THOMAS'S HOSPITAL.

#### FRACTURE OF THE SEVENTH CERVICAL VERTEBRA. *

Cases of fractured spine used to be considered as something uncommon in the pages of a journal, but now, thanks to the "gentlemen connected with the press," as Sir Richard Birnie has it, they are becoming plenty as black-berries.

H. C. æt. 35, received an injury to the spine, on the 1st of December, from a sack of flour falling from a height of 10 feet upon his shoulders. He was stunned by the blow, and lost the use of his lower extremities, but did not enter the hospital until the 5th. The lower extremities, at this time, were quite paralyzed, but he could raise his arms to a certain extent —breathing diaphragmatic—no priapism —pain on attempting to move his head. No displacement of vertebræ could be discovered, so that Mr. Green was disposed to consider the symptoms as dependent on effusion. Cupping—injections, and the catheter three times a day, were employed, but the urine in a few days became decidedly ammoniacal and offensive, sloughs formed on the nates and sacrum, and on the morning of the 23rd the poor fellow died.

*Dissection.* The arch of the 7th cervical vertebra was found to be broken, its right inferior oblique process dislocated forwards to the extent of a quarter of an inch, and its body fractured across. Upon the theca beneath the first dorsal arch there was a coagulum of blood, whilst the membrane here was vascular and distended. On exposing the medulla, much serous fluid escaped, and the marrow itself opposite the last cervical vertebra was of the consistence of cream.

This softening of the medulla Mr. Green, in his clinical lecture, very properly considered as dependent on the injury (and consequent inflammation) of its substance, rather than on any pressure exercised by the bone. Mr. Green also observed that, on the 8th day after the patient's admission there were signs of returning sensation in the lower extremities, the patient being able to distinguish which foot was touched; but on the following day these good symptoms had disappeared. This, Mr. Green was disposed to attribute to the setting up of inflammatory action, which induced a temporary excitement in the spinal marrow, and temporary return of sensibility in consequence. The reporter, whilst he appears to be struck by this "ingenious explanation" of Mr. Green's, (an explanation, by the bye, which was given long ago by Mr. Charles Bell, in his celebrated controversy with Sir Astley Cooper,) declares pretty plainly, that Mr. G. laboured under an "illusion" as to the *fact* of the amendment having taken place on the day mentioned. The reporter examined the man soon after Mr. G. and found, "to his complete satisfaction, that *there was no return of sensibility*." As the shock to Mr. Green's nervous system must be very severe indeed upon reading this, the reporter has very judiciously and humanely endeavoured to allay the constitutional irritation, by declaring that what he did, was only "with a view of establishing truth," and preventing the dissemination of so dangerous a doctrine, as that H. C. could tell which toe was pinched upon the eighth day!

---

### 5. ST. BARTHOLOMEW'S.

#### MALIGNANT BLEEDING POLYPUS OF THE NOSE.

An interesting case of this terrible disease is detailed in No. 6 of the Gazette. We must refer our readers for the details, which are exceedingly well given, to the journal in question; but we may just

* Lancet, No. 228, Jan. 12.

state, that it had existed for eighteen months, had been removed by the forceps twenty-three different times, and was constantly bleeding. After putting the patient under proper diet, &c. Mr. Earle attempted to dissect it out by an incision, commencing at the top of the right nasal bone, and extending through the ala nasi. The polypus was attached to the nasal and maxillary bones, which were rough, and their Schneiderian membrane thickened. Each stroke of the knife was followed by profuse hæmorrhage, and it was thought necessary to remove part of the nasal and maxillary bones, and to apply the actual cautery. The operation, which does no discredit to Mr. Earle, was performed on the 29th September, and, by the 30th October, the fungus had again made its appearance. It went on increasing in size, bled profusely, and, on the 30th Dec. the patient died.

On dissection, the fungus appeared to have taken its origin from the membrane covering the vomer, superior turbinated bone, and cribriform lancella of the æthmoid. The lungs, the liver, the spleen and pancreas, the renal capsules, fat surrounding the bladder and rectum, &c. &c. were studded with tumours, some of them medullary, some of them resembling clots of blood, and readily breaking down under the fingers.

### Strangulated Exomphalos in the Eighth Month of Pregnancy.*

E. Wright, æt. 31, had been always subject to a tumour at the navel, which, about the 4th or 5th month of pregnancy, invariably swelled and became painful, but subsided under the use of a purgative. On the 30th Sept. being then 7 or 8 months gone with child, she was seized with pain in the umbilicus and vomiting, which, not being relieved by castor oil, she entered the hospital, on the 1st Oct. under the care of Mr. Lawrence. The hernial tumour was soft, irregular, not very tender, and consisted principally of omentum, whilst there was no tenderness whatever of the abdomen. Castor oil, leeches, taxis, &c. were employed in vain, and Mr. Lawrence, notwithstanding the mildness of the symptoms, determined on operating. On laying open the sac, a quantity of omentum, and a small portion of discoloured intestine, were exposed. The latter was returned, and a part of the omentum removed, one or two small vessels requiring a ligature. She had four or five motions after the operation, but, next morning, there came on inflammation about the wound, for which she was bled to sixteen ounces—had twenty leeches to the abdomen, &c. On the 3d, the bleeding and leeches were repeated, and a poultice applied to the wound. *Oct. 4th.* Better. V.S. ad ℥xiv. An abscess formed "on the tumour," (this means, we suppose, in the omentum) which was opened, and the patient did very well.

It is not many years since recovery from the operation for hernia, at *some* of our hospitals, was no very common occurrence, whilst at present, when circumstances are tolerably favourable, patients as rarely sink under or after it. This change in the comparative results of the operation, we have no hesitation in ascribing to the circumstance of surgeons resorting to it much earlier than they used to do. In the case, however, under review, we are not quite sure that the protrusion might not have been returned without the operation, for the symptoms were exceedingly mild, and the reporter makes no mention whatever of the state of the stricture. It may have been firm, it may have been lax, or there may have been no stricture at all, from any thing which appears to the contrary on the face of the report, although the direction of the cuts, the sutures, and the straps, are detailed with the most laudable fidelity. We observe that in this, as in most other instances, Mr. Lawrence removed a portion of the omentum, and certainly, as far as the case goes, without any ill effect. We should be glad to hear the results of Mr. Lawrence's experience on this point, as it is one of much interest, and a good deal of difficulty.

---

### College of Physicians *v.* Dr. Harrison.

We were right in our information, that a committee had been appointed to collect proofs of Dr. Harrison's practice. They have effected their purpose (as they be-

* Lancet, No. 228.

lieve) and notice of action has been served. We said that, had we been in Dr. Harrison's place, we should have given up the admission of practice at once, and thus met the action boldly. Dr. H. by halting in the commencement of the battle, has lost the "vantage ground," as far as concerns himself personally, without gaining any adequate security of position by this ill-timed caution. This, however, makes no difference in the general result of the contest. Nothing will give us greater pleasure than the fact of a verdict being gained by the College. It is just what we ardently hope for. While an unjust law slumbers, it is safe. The moment it is put in execution, the process of abrogation commences. A statute originally designed for the suppression of quacks, is now turned against regular physicians of the first medical university in the united kingdom. We had little idea that the council of the College would, in the year of our Lord, 1828, prove the truth of the ancient adage—

"Quem Deus vult perdere, prius dementit."

Anxious for the establishment of LIBERAL INSTITUTIONS and JUSTICE, we shall restrain ourselves on the present occasion, lest we should disturb the natural course of events. Mr. Brougham will remove the veil, and the picture which will be drawn of the state of this branch of the profession, in a court of law, will open the eyes of statesmen—while the general indignation of the Profession, at such a flagrant insult to the diplomas of the Edinburgh, and other regular universities, will lead to the desired object—a general investigation of the state of the profession.

---

### LIBERALITY AND ILLIBERALITY.

We were much grieved to see a letter, lately published in the MEDICAL GAZETTE, from a correspondent of Dr. A. T. Thompson, reflecting, in rather illiberal terms, on foreign schools of medicine. Those who know how openly and hospitably English students and practitioners are received in the public schools and institutions of the Continent, cannot but deplore the impolicy and narrow-mindedness of individuals, who, from hasty and crude views of things, permit themselves to injure their countrymen, and the whole of the English medical profession, in the eyes of liberal foreigners, by such prejudiced and unfounded statements as those alluded to.

---

But what shall we say to the disgraceful scenes that are now exhibiting in the medical press, in respect to the injuries sustained by the medical officers of public institutions from the misrepresentations of FALSE REPORTERS? Let any man read the deluges of abuse which have been poured on Mr. Earle and Mr. Keate, in the last Number of the LANCET, for having merely corrected the wilful falsehoods of ignorant reporters, and say whether or not the SATURNIA REGNA are returning in this country! The Lancet is playing a desperate game. It seems determined to try how far the most vulgar ribaldry and Billingsgate slang can be crammed down the throats of a learned and liberal profession, as a substitute for knowledge, candour, and practical information. Thus, whole columns of that journal are filled with the most low-lifed and disgusting epithets, lavished on men of acknowledged probity, talents, and honour, while all their statements are distorted, garbled, and misrepresented, so as to make a few of the very lowest and most ignorant of the profession laugh, and all the sensible and ingenuous grieve—if they can permit themselves to wade through such detestable trash! We confess, however, that we are not sorry to see justice, good sense, and proper feeling, thus *violently* outraged. It is precisely in this way that evil works its own ruin. Whatever may be the proportion in which good and bad principles are mixed in human nature, a time always arrives, when the former is approved, and the latter spurned. That period is rapidly approaching, if we be not much mistaken. At all events, the advocates of the evil principle are taking the most direct paths to bring about the triumph of the good. We would advise the advocates of the LATTER to oppose a calm, but steady resistance, in the detection of error and misrepresentation, trusting to the good sense of Englishmen, and the justice of their cause, for a final and signal victory.

"When Peace and Mercy, banished from the plain,
Sprang on the viewless winds to Heaven again---
All --all, forsook the friendless, guilty mind---
But HOPE, the charmer, lingered still behind!"

